# Sensitivity to the acoustic correlates of lexical stress and their relationship to reading in skilled readers

**DOI:** 10.2478/v10053-008-0122-0

**Published:** 2012-11-16

**Authors:** Gareth J. Williams, Clare Wood

**Affiliations:** 1 Division of Psychology, Nottingham Trent University, United Kingdom; 2 Department of Psychology and Behavioural Sciences, Coventry University, United Kingdom

**Keywords:** lexical stress, multisyllabic words, reading, comprehension

## Abstract

The role of suprasegmental information in reading processes is a growing area of interest, and sensitivity to lexical stress has been shown to explain unique variance in reading development. However, less is known about its role in skilled reading. This study aimed to investigate the acoustic features of suprasegmental information using a same/different cross-modal matching task. Sixty-four adult participants completed standardized measures of reading accuracy, reading speed, and comprehension and performed an experimental task. The experimental task required the participants to identify whether non-speech acoustic sequences matched the characteristics of written words. The findings indicated differences in responses depending on where the lexical stress was required for the word. Moreover, evidence was found to support the view that amplitude information is part of the word knowledge retrieval process in skilled reading. The findings are discussed relative to models of reading and the role of lexical stress in lexical access.

## Introduction

Reading is primarily a linguistic activity and therefore spoken language and reading share similar skills. Furthermore to date, the speech-based skill that has been most consistently studied in relation to reading is that of sensitivity to segmental phonology. However, spoken language comprises a range of other acoustic signals (such as prosody and lexical stress) that have, until recently, received far less attention. One difference between speaking and reading is that a typical speaker will be able to modify the tone and amplitude of a word (some of the suprasegmental properties) for the benefit of the listener but, as words on a page in English do not explicitly have this information, it is likely that a reader will have to produce the tone and amplitude information based on prior experience of reading and speech. However to date, there have been few studies that have investigated whether this is the case. This study aimed to investigate skilled readers’ sensitivity to the tone and amplitude patterns that have been derived from words and then investigate the relationship between this sensitivity and standardised skills in reading.

In the dual route model of reading (Coltheart, Rastle, Perry, Langdon, & Ziegler, 2001), known words are subject to a lookup a lexical lookup. The lookup for print is initially orthographic and then phonological and both lookup lexicons interact with each other. Once a word is identified, it can then proceed to being read aloud. In applications of the dual route model to multisyllabic words Rastle and Coltheart (2000) have argued that for known words, the suprasegmental properties would be applied using some form of lexical lookup. Connectionist models of multisyllabic reading have suggested a similar process for known words (Perry, Ziegler, & Zorzi, 2010). It is possible that speech perception research offers an insight into how suprasegmental information would be involved in this lexicon. Cutler and Norris (1988) showed that cues in the syllable stress of a word might be the initial trigger for a listener’s lexical lookup when a word is spoken. In this context, a stress cue for the word *information* would be the emphasis a speaker places on the *ma* in the word. This emphasis is regarded as suprasegmental because it is not a direct part of the segmental phono-logy of the word.

Yet it is possible that sensitivity to general changes in emphasis, expressed in amplitude and tone frequency, may also contribute to reading performance in other ways. For example, in order to differentiate between the verb and nouns of words such as *record*, tone and amplitude information would need to be represented and processed. Such information has to be represented by the skilled reader just as much as text needs to be decoded at a segmental level. By implication, it would seem likely that skilled readers need to be sensitive to lexical stress changes and be able to relate such information to the words that they are reading, even when they are reading silently. For example, Ashby, Treiman, Kessler, and Rayner (2006) found evidence that silent adult readers are sensitive to the suprasegmental properties of words in an eye tracking study.

Furthermore, there is evidence that suprasegmental patterns can be encoded in the orthographic patterns of words (Kelly, 2004), and the development of assigning these patterns is likely to be based on the statistical regularities of the patterns from reading (Arciuli, Monaghan, & Ševa, 2010). Print experience may be one way of developing sensitivity to suprasegmental patterns that can be applied to reading. It is possible that auditory information and speech perception may also facilitate this sensitivity. If so, then it should be possible to detect this sensitivity in skilled adult readers and to show that it may account for some significant variation in reading performance.

The presumption that auditory processing, and speech perception skills in particular, may underpin phonological sensitivity has been confirmed in a number of studies, for example in reading skills (Mann & Foy, 2007) and reading difficulties (Brady, Shankweiler, & Mann, 1983; Breier, Fletcher, Denton, & Gray, 2004; Manis, McBride-Chang, Seidenberg, & Keating, 1997; Metsala, 1997), although the evidence from McBride-Chang (1996) suggested that speech perception indirectly contributes to reading skills through phonology. Whether auditory processing may contribute to other aspects of reading has received less attention.

In reading, segmental phonology may be linked to speech perception but both abilities could be linked to fundamental skills in general auditory processing and rapid auditory processing in particular (Farmer & Klein, 1995; Reed, 1989; Tallal, 1980, 1984; Tallal, Miller, Jenkins, & Merzenich, 1997). For example, speech and auditory systems can pro-cess rapidly changing information such as the tonal frequency changes for b and d. It would follow that much of the suprasegmental information would be made up of changes in rapid acoustic information rather than strictly the phonological information. Although the idea that a deficit in rapid auditory processing can explain reading difficulties has been largely rejected (e.g., Bretherton & Holmes, 2003; Marshall, Snowling, & Bailey, 2001; Mody, Studdert-Kennedy, & Brady, 1997), the idea that more fundamental auditory processing skills may contribute to phonological sensitivity and reading ability has been recently revisited (e.g., Thomson, 2009). It is suggested, for example, that an individual’s sensitivity to changes in amplitude across a word (amplitude envelope perception or “rise time”) may be a characteristic of children with reading difficulties (Goswami, Gerson, & Astruc, 2010).

Although amplitude and, to some extent, tone frequency have been studied in relation to reading, considerably more research has investigated the role of amplitude, frequency, and also duration in spoken word recognition. Perceived differences in the duration of syllables or single syllable words have often been shown to be a factor in English (Klatt, 1976) and Chinese (Shen, 1993) speech perception. Participants have also found it easier to extract duration information from speech compared to amplitude information (Turk & Sawusch, 1996). However, Fry (1955) found that, although duration information allowed participants to distinguish between primary and secondary syllables, it was amplitude differences between first and second syllables that informed participants about whether an ambiguous two syllable word (e.g., *record*) was a noun or a verb. Moreover Plag, Kunter, and Schramm (2011) argued that, even if duration was distinguishable between two syllables in speech, its role was still unclear. It might be that duration plays a role in the differences between changes in stress between syllables in a word and changes between stress in syllables in a sentence. Bettagere (2010) found that the duration of a syllable was the strongest differentiator of a lexical stress that is placed on a syllable in a word compared to where an emphatic stress is placed on a word in a sentence to clarify the sentence’s meaning.

In contrast to amplitude and duration information in words and sentences, the changes to tone frequency, initially raised as a possible factor in speech perception and production by Bolinger (1958), have been shown to have an important role in distinguishing suprasegmental information by Lieberman (1960) and by Morton and Jassem (1965). Both researchers found that variations in the frequency between syllables in a word influenced listener judgments about stress patterns. However, frequency has a complex relationship to speech. Shattuck and Klatt (1976) found that /*ab*/ and /*ba*/, although appearing to be mirrors of each other in speech, do not have equivalently mirrored frequency changes. Moreover Bettagere (2010) found that speakers do not often vary the frequency of their speech in stress patterns.

Other factors may affect a listener’s sensitivity to amplitude, frequency, and duration. Researchers have noted a “trading effect,” in that if a stress pattern cannot be distinguished by one acoustic correlate, it can often be distinguished by another (Lieberman, 1960; Lyberg, 1979; Mcclean & Tiffany, 1973; Morton & Jassem, 1965; Turk & Sawusch, 1996). Moreover, the speech perception of acoustic correlates is mediated by how soft or loud the speech is overall (Mcclean & Tiffany, 1973) and by the speech rate (Morton & Jassem, 1965). Where a stress is placed may depend on the role a word plays in a sentence, for example, whether it is the subject or the object. This is especially the case in sentences where these words have an ambiguous role (Bettagere, 2010; Snedeker & Trueswell, 2003). Moreover, which acoustic correlate is important depends on which syllable has the stress placement (Mcclean & Tiffany, 1973). Furthermore, suprasegmental patterns are subject to considerable variation between speakers (Behrens, 1988) and so a listener would need to be accommodating of these differences in processing speech. Overall, the research in speech perception underlines that a typical adult reader will bring considerable experience of suprasegmental information with them when he or she engages in other linguistic activities such as reading.

One way of investigating suprasegmental properties is to use non-speech stimuli. Many studies use non-speech tones as stimuli to study aspects of these fundamental auditory processing skills. Richardson, Thomson, Scott, and Goswami (2004) found that, in tasks that required auditory perception of amplitude changes in non-speech stimuli, children with dyslexia showed difficulties compared with typical children and that their abilities at the non-speech tasks were associated with phonological skills. The researchers suggested that children’s sensitivity to the signals that provide information about speech units, such as phonemes and syllables, help facilitate subsequent phonological sensitivity. The findings have been supported by further research with typical children, individuals with dyslexia and also by cross linguistic studies (see Corriveau, Pasquini, & Goswami, 2007; Goswami et al., 2002, 2010; Richardson et al., 2004; Thomson, Fryer, Maltby, & Goswami, 2006). Additional research would help complement these findings by exploring the sensitivity to suprasegmental cues in skilled adult readers.

This study therefore related such sensitivity to adults’ performance on three different reading measures. With others we assumed that *skilled reading* may be viewed as a combination of accurate and fast reading that contribute to reading comprehension (cf. Brooks, Everatt, & Fidler, 2004). Moreover, when assessing the nature of the contribution of auditory sensitivity to these various reading measures, we also considered the participants’ performance on vocabulary in the analysis of reading skills (e.g., see Braze, Tabor, Shankweiler, & Mencl, 2007; Gathercole, Willis, Emslie, & Baddeley, 1991; Wise, Sevcil, Morris, Lovett, & Wolf, 2007).

To assess sensitivity to amplitude and tone frequency in the context of reading, a variation on a matching task was used. Participants were presented with an auditory stimulus, which either matched or did not match a target word in terms of its pattern of amplitude or frequency changes. The target word was then presented on screen and participants were asked to indicate whether the initial auditory stimulus matched or did not match the suprasegmental pattern of the word presented on screen. If participants heard an auditory stimulus congruent to the visual word such that their reaction time was faster and they were more sensitive in detecting this match (compared with where the auditory stimulus and word were not matched) then this would indicate that information relating to amplitude or tone frequency was encoded in the lexicon.

The experimental task was designed in order that participants would make a decision about the lexical stress pattern of the target word in relation to either its amplitude or frequency properties. The initial acoustic sequence acted as a cue that was stored in memory until a matching judgment could be made. The target word that was subsequently displayed, as text, was then processed by following the lexical access route of the dual route in reading. As the word was a real word, both orthographic and phonological lexicons were subject to a look-up process and the lexical stress pattern of the word was then applied prior to the judgment. The judgment that the participant made was based on whether the previously stored cue matched or did not match features of the lexical stress pattern.

There is evidence from linguistic research that an approach of this nature offers an appropriate method of investigation; however the findings are mixed. In one study, Dutch spoken syllable fragments were manipulated by amplitude to be congruent or incongruent with the word stress of a later visually presented word (van Donselaar, Koster, & Cutler, 2005). The researchers found faster reaction times for congruent pairs of primes and targets than for incongruent pairs. Cooper, Cutler, and Wales (2002) found an effect with a fragment priming design, particularly for two syllable words. In contrast for picture naming of two-syllable words where the first syllable was louder (strong) and the second was lighter (weak) such as the noun for *record*, these words had faster responses compared with weak-strong words. However broadly, researchers have found little evidence of faster, more sensitive, responses for suprasegmental word information and picture naming (Schiller, Fikkert, & Levelt, 2004). One possibility is that the words used in these studies were too short for effects to become pronounced, as suprasegmental information becomes particularly important in longer multisyllabic words as these words would contain more information. Consequently, multisyllabic words longer than two syllables were selected for use in this study. Finally, as the numbers of phonemes in a word can also affect reading processes (Spencer, 1999), this property would also need to be taken into account in selecting target words.

The aim of this study was to investigate the role of two aspects of suprasegmental information in words, namely the changes in amplitude and tone frequency, and then investigate their relationship to reading speed, reading accuracy, and comprehension. In particular, we wanted to know whether participants’ reaction times on the experimental task would be affected by pair type and the pair congruency of target words. This would provide an index of the relationship that pair matches have to the target words in the mental lexicon, faster correct responses to matches suggesting a closer relationship between amplitude and/or tone frequency and the target word. We also wanted to calculate each participant’s sensitivity (*d*’) to each of the different auditory pair types for comparison and to consider which acoustic feature they were most sensitive to. Finally, we also wished to know whether the sensitivity scores could predict performance on measures of reading accuracy, reading speed, and comprehension.

## Method

### Participants

Sixty-four adult undergraduate students (six males and 58 females) from two universities in the Midlands of the United Kingdom took part. All were native English speakers. The mean age of the group was 21 years (*SD* = 3.85). Participants provided self-reports of difficulties with reading, hearing, and any additional languages that participants could speak. Given the very few reports of difficulties and the generally high reading level, no participants were excluded from the study based on their self-reports. A summary of the baseline measures for the participants is reported in Table 1.

**Table 1.** Means and Standard Deviations for the Reading Measures (*N* = 64)

Measure	*M*	*SD*
Reading speed^a^	64,83	23,30
Reading accuracy^a^	61,64	21,84
Reading comprehension^a^	69,92	20,27
Phonological awareness^b^	13,48	3,50
Nonword repetition^a^	34,86	21,00
RAN^a^	39,83	28,36
Vocabulary^c^	44,98	13,36

^a^percentile. ^b^raw/18. ^c^*t*-score.

### Materials

#### Reading accuracy, reading speed, and comprehension

This measure, the Adult Reading Test (Brooks et al., 2004), comprised passages of around 300 words that were read aloud. Three measures were taken. Participants’ reading accuracy was scored on the number of mistakes made in reading the passage, and the accuracy score was calculated in line with the manual instructions. The time taken to read each passage was recorded as a measure of reading speed and then calculated in words per minute (wpm). Finally comprehension questions were asked about the passage that had just been read. The questions consisted of facts about the passages, questions from memory, and inference questions. Participants were not allowed to look back at the passage when answering the comprehension questions. For the comprehension questions, one point was awarded for each correct response. Five passages were read by each participant and the test was administered according to the standardized instructions. For this sample, the internal reliability coefficients (Chronbach’s ) for accuracy and comprehension were .73 and .75, respectively.

#### Phonological Awareness

This measure (Chronbach’s = .86) was taken from the Comprehensive Test of Phonological Processing (CTOPP; Wagner, Torgensen, & Rashotte, 1999). Participants were asked to repeat a word with a specified syllable or letter-sound missing, but two items were removed as they were not phonologically appropriate for a British English accent (these were *faster* and *slick*). The number of correct responses was recorded as a measure of this task.

#### Nonword Repetition

This measure (Chronbach’s = .50) from CTOPP (Wagner et al., 1999) was used to assess whether verbal memory was associated to the ability to respond to the matching task as participants were hearing a four tone auditory stimulus and then responding to a target word. Participants were asked to repeat a non-word (e.g., *jup*) that was played to them on a CD and, as the task progressed, the length of the non-word items increased (e.g., *viversoomouj*). The number of words repeated successfully was recorded as a measure of this task.

#### Rapid Automatised Naming

The Rapid Automatised Naming task (letters) from the CTOPP (Wagner et al., 1999) was used to assess speed of phonological retrieval. After ensuring that participants were able to read aloud the target six letters individually, participants were presented with a four by nine grid array of letters (e.g., *s t n a k c*…) and were asked to name aloud the letters as quickly as possible. Participants’ total time to complete two arrays, in seconds, was recorded as the measure for this task.

#### Vocabulary

This measure (Chronbach’s = .84) was the vocabulary subtest from the Wechsler (1999) Abbreviated Scales of Intelligence. Participants were asked to provide the definitions of a words (e.g., of *map*) and the quality of the response, in line with the manual’s guidelines, was scored with a 2, 1, or a zero.

#### The Suprasegmental Matching Task

It is possible for syllables to have one of three types of stress pattern that make up the suprasegmental information of a word: primary, secondary, or “weak” (unstressed). For example, in the word *respiration*, the first syllable *res* carries secondary stress, the second syllable *pir* is unstressed, the third syllable *a* has primary stress, and the final syllable *tion* is unstressed. In this study, 24 four-syllable words (see Appendix A)were selected for the suprasegmental task. Four-syllable words were used so that there was sufficient lexical information in the target word.

As would be expected, four-syllable words may have a primary stress on the first, second, third, or fourth syllable. Schiller, Jansma, Peters, and Levelt (2006) noted that words of more than one syllable often have a default primary stress. This default stress could be applied by rule and not necessarily encoded in a lexicon. In their study they noted that for Dutch two-syllable words, the default stress is on the first syllable. This is also the same for English (Kelly, 2004). However, Schiller et al. (2006) found that Dutch three-syllable words are not likely to be susceptible to a default stress as there was a more even distribution of stress in the first, second, and third syllables both by type and by token. To assess whether four-syllable words were susceptible to default stress effects in English, the 9,637 four-syllable words with phonetic records were extracted from the English Lexicon Project (ELP) database (Balota et al., 2007) and these words were then categorized, based on the phonetic records, as having a primary stress on the first, second, third, or fourth syllable. Few words had a fourth syllable stress (type = 0.8%,token = 0.8%), stress patterns were otherwise fairly evenly distributed in the remaining three word categories suggesting that there would be little likelihood of default rule effects: second syllable (type = 41.1%,token = 44.4%), third syllable (type = 28.5%, token = 34.3%), and first syllable (type = 29.6%, token = 21%). Words with a primary stress on the third syllable (secondary-unstressed-primary-unstressed [2u1u]), and words where the primary stress was on the second syllable (unstressed-primary-unstressed-unstressed [u1uu]) were used as they were representative of suprasegmental patterns that individuals encounter in text and speech. Twenty-four words were selected, 12 with a 2u1u syllable stress pattern (e.g., *education*) and 12 with a u1uu syllable stress pattern (e.g., *capacity*).

For the matching task, for each group of 12 words half had congruent pairs whilst half had incongruent pairs; for instance, if a congruent a word such as *economic* would have a 2u1u cue while if incongruent, it would have the lexically plausible u1uu pattern as its cue. Although the same word lists were used for each condition, the congruent or incongruent pairings were different for each condition but consistent for each participant. The stress pattern and the number of phonemes of the words for British speakers was verified using data from Roach, Hartman, and Setter (2006).

Phoneme length was counted based on data provided by Wilson (1987) and the word groups were controlled so that there was no significant difference for phoneme length. There were no significant differences comparing either group of words for word length, *t*(22) = 0.64, *p* = .53, *d* = 0.27; phoneme length, *t*(22) = 1.55, *p* = .14, *d* = 0.62; word frequency based on Kucera and Francis (1967), *t*(22) = 0.43, *p* = .67, *d* = 0.18; British National Corpus (Leech, Rayson, & Wilson, 2001), *t*(22) = 0.04, *p* = .97, *d* = -0.01; or ELP (Balota et al., 2007), *t*(22) = -0.83, *p* = .42, *d* = -0.35. In general, the effect sizes (*d*; Cohen, 1992) were small and this was as intended so that the two lists were comparable. The only exception was phoneme length where the difference was non-significant but there was a medium effect size. Table 2 summarizes the results for both word groups.

**Table 2.** Means and Standard Deviations for Word Frequency and Phonemes for Word Categories in the Experimental Task

	Word categories			
	2u1u (*n* = 12)		u1uu (*n* = 12)	
	*M*	*SD*	*M*	*SD*
KF word frequency	137,50	73,90	125,58	62,02
BNC word frequency	156,67	102,11	158,08	86,95
ELP word frequency^a^	10,53	0,52	10,77	0,85
Word length	9,92	1,00	9,58	1,51
Phonemes	9,08	0,67	8,67	0,65

*Note*. BNC = the British National Corpus. EL*p* = the English Lexicon Project. KF = Kucera Francis (cf. Kucera & Francis, 1967). ^a^HAL log frequency

The experiment had two conditions (as blocks) relating to the two ways under investigation in which suprasegmental information can be carried by a multisyllabic word, by amplitude or tone frequency. Rather than use manipulated spoken words, non-speech stimuli were designed comprising sequences of four tones.

The amplitude condition’s auditory sequences had four tones of 500 Hz frequency with a 200 ms duration and with a 5 ms interstimulus interval. A primary syllable had an amplitude at 100% of a word sequence’s maximal volume, an unstressed syllable had an amplitude of 25% of the volume whilst an secondary syllable had an amplitude midway between the other two of 62.5%. The tone frequency condition cues had a 200 ms duration, a 5 ms interstimulus interval, and had an amplitude at 100% of the sequence’s maximal volume. In this condition, the primary syllable had a frequency of 600 Hz, the unstressed syllable had a frequency of 400 Hz, and the secondary syllable had a frequency of 500 Hz. The Chronbach’s for the task was .58.

### Procedure

Participants sat in front of a computer at a distance of around 50 cm from the screen and the tasks were presented using Superlab 4 (Cedrus Corporation, 2007). The auditory stimuli were presented through headphones at a sound level comfortable for the participants in a sound attenuated room. Participants were asked to use their preferred hand to operate the mouse and instructed to place their index finger on the left button and their middle finger on the right mouse button. They were informed that they would hear a sequence and then see a word, they were to press the right mouse button if the sequence and word did not match, and the left mouse button if the two did match.

For the task, a fixation point was presented for 1,000 ms. Next, the auditory sequence was presented. Each auditory sequence was 820 ms in duration. Immediately after the auditory sequence was presented, the target word would appear on screen in white text, 36 point Tahoma, on a black background, and remain visible until the participant pressed a mouse button. There was then a 1,000 ms intertrial interval before the next fixation point was presented.

Response times and detection sensitivity were measured by the suprasegmental matching task. For response times, only correct responses were used and outliers were addressed in line with recommendations for reaction time studies (Ratcliff, 1993). Outliers were considered as any participant’s reaction time 2.5 *SD* above the mean or shorter than 150 ms, and scores found meeting this criteria were replaced with the mean for the pair type. In this way, 2% of the responses were replaced across the two pair types. The pattern of significant results of the response time analyses remained the same for the data when the raw response times were log transformed and outliers 2.5 *SD* above or below the mean were replaced with the mean for the pair type, consequently only the analyses based on the raw response times are reported in the results section. For detection sensitivity, *d*’ was calculated based on the hits and false alarms that participants made to the pairs of congruent and incongruent pairs. In line with Macmillan and Creelman (2004), where participants had proportions of hits to false alarms that resulted in scores of zero or one, these scores were replaced with 1/(2*N*) and 1 - 1/(2*N*) respectively, where *N* is the number of responses. There were five replacements of zeroes and three replacements of ones across all of the responses.

Prior to the experimental tasks, participants were familiarized with the task in an activity that explained the word stress task and then provided feedback with a series of sample trials. The participants heard example auditory sequences paired to a word that was not in the experimental task. There were six pairs presented and once the familiarization block had been completed, participants began the experimental tasks. There were two blocks in the word stress experimental task, one block where amplitude was manipulated and one where tone frequency was manipulated. Whether a target word was paired with a congruent or with an incongruent auditory sequence was selected pseudo-randomly (with the constraint that half of the trials were congruent and the other half incongruent) by the computer. The participants heard each auditory sequence and target pair once in each block. With 24 target words, this resulted in 48 auditory sequence and target pair trials for each participant. The order of the suprasegmental matching task and the reading measures were counterbalanced across participants.

## Results

The initial aim was to consider the role of sensitivity to the auditory sequences and the word targets. Sensitivity was measured in two ways: reaction time and *d*’. The first set of analyses investigated how reaction times were affected depending on whether the participants heard amplitude or tone frequency patterns at the start of the pair of stimuli or whether the pairs were congruent or incongruent.

### Response times comparing pair type and congruency

In the response time analysis, 59% of the responses were correct and in order to confirm that this was above chance a one-sample t-test, using the overall *d*’ scores, was carried out. The *d*’ score was compared to a value of zero as this value, in detection theory, would represent that participants were guessing (Macmillan & Creelman, 2004). The results indicated that the participant responses were above chance (*M* = 0.47, *SD* = 0.56), *t*(63) = 6.67, *p* < .01, *d* = 0.83 (two-tailed).

To investigate the differences in response times for the two types of pairs in the experimental task, a 2 Pair Type (amplitude, tone frequency) × 2 Congruency (congruent, incongruent pairs) ANOVA was carried out. There was a main effect of pair type, *F*(1, 60) = 7.46, *MSE* = 442010.35, *p* < .01, partial η^2^ = .11, where there was a significantly faster response to tone frequency pairs (*M* = 1952.0 ms, *SD* = 755.5) than amplitude pairs (*M* = 2184.4 ms, *SD* = 976.1). However, there was no significant main effect of congruent (*M* = 2028.3 ms, *SD* = 862.3) compared with incongruent pairs (*M* = 2108.1 ms, *SD* = 816.1), *F*(1, 60) = 1.81, *MSE* = 213969.35, *p* = .18, partial η^2^ = .03. There was no significant interaction, *F*(1, 60) = 0.33, *p* = .57, partial η^2^ = 0.

### Sensitivity to amplitude and tone frequency

To investigate how sensitive to matches participants were for the two conditions, amplitude and tone frequency, a paired t-test was carried out. There was no significant difference between amplitude (*M* = 0.16,*SD* = 0.45) and tone frequency (*M* = 0.04, *SD* = 0.41) sensitivity, *t*(63) = 1.57, *p* = .12, *d* = 0.28 (two-tailed), and the effect size was small, which would suggest participants had similar levels of sensitivity to both conditions.

### Item analysis

A series of two item analyses was carried out, one for response times and a second for accuracy. For response times, only correct responses were used and there was no significant main effect of pair type for amplitude (*M* = 2097.5 ms, *SD* = 169.7) compared to tone frequency (*M* = 2174.9 ms, *SD* = 256.5), *F*(1, 22) = 1.67, *MSE* = 42902.57, *p* = .21, partial η^2^ = .07. However, there was a main effect for suprasegmental pattern: u1uu patterns (*M* = 2068.0, *SD* = 124.8) were responded to significantly faster than 2u1u patterns (*M* = 2204.4, *SD* = 173.9), *F*(1, 22) = 4.87, *MSE* = 223058.42, *p* < .05, partial η^2^ = .18. There was no significant interaction, *F*(1, 22) = 0.02, *MSE* = 653.35, *p* = .90, partial η^2^ = 0.

In order to investigate the accuracy rates of items relative to the lexical stress patterns, a by-items 2 Pair Type (amplitude, tone frequency) × 2 Suprasegmental Pattern (u1uu, 2u1u) ANOVA was conducted. There was a significant main effect for pair type, *F*(1, 22) = 4.47, *MSE* = 0.02, *p* = .046, partial η^2^ = .17, tone frequency items (*M* = 0.62, *SD* = 0.15) had a higher accuracy overall rate than amplitude (*M* = 0.55, *SD* = 0.08). There was no significant main effect for suprasegmental pattern, *F*(1, 22) = 0.62, *MSE* = 0.02, *p* = .44, partial η^2^ = .03, and there was no significant interaction, *F*(1, 22) = 0.02, *p* = .90, partial η^2^ = .01. In both pair types no one word accounted for a large proportion of the errors. The word with the highest error proportion for amplitude was *philosophy*, accounting for 5.1% of the errors, and for tone frequency it was *particular*, accounting for 5.3% of the errors.

### The contribution of amplitude and tone frequency sensitivity to adult reading

Prior to addressing the relationship between amplitude and tone frequency sensitivity and the reading measures, it was possible that differences in sensitivity were due to individual differences in short-term memory. However, there were no significant associations between verbal memory (as measured by nonword repetition) and the matching task measures. The correlations between the sensitivity measures and nonword repetition are reported in Table 3.

**Table 3.** Zero Order Correlations Comparing Standardised Measures with Amplitude and Tone Frequency (*N* = 64)

	2	3	4	5	6	7	8	9
1. Reading speed (wpm)	.314*	.059	.156	.301*	-.101	.260*	.189	.019
2. Reading accuracy (raw)		-.537**	.128	.170	.096	.204	.058	-.048
3. Reading comprehension (raw)			-.269*	.042	-.270*	.346**	-.005	-.025
4. Phoneme elision (raw)				.007	-.112	-.339**	-.217	.141
5. Nonword repetition (raw)					-.114	.442**	.066	-.030
6. RAN seconds						.067	-.009	.004
7. Vocabulary (raw)							.339**	.064
8. Amplitude *d*’								-.007

*Note*. wpm = words per minute. *p < .05. **p < .01.

The contribution of the sensitivity scores to the different types of pairs for the three Adult Reading Test measures (raw scores for reading accuracy, speed, and comprehension) was the final aim of the study. The raw scores for the speed, accuracy, and comprehension measures were each taken in turn as the outcome variable and forced entry multiple regression models were produced. Zero order correlations were carried out for the sensitivity measures and the reading, phonological awareness, rapid automatized naming, and vocabulary measures, using raw scores. As Table 3 indicates, while tone frequency and amplitude sensitivity did not correlate significantly with any of the reading measures, vocabulary was found to be correlated positively with sensitivity to amplitude pairs. Vocabulary itself was also correlated with reading speed, reading comprehension, phonological awareness, and nonword repetition. When sensitivity to amplitude pairs was partialled out (see Table 4), the significant correlation between reading speed and vocabulary disappeared. However when partialling out tone frequency (see Table 5), the pattern of significant correlations between vocabulary and reading speed, reading comprehension, phonological awareness, and nonword repetition remained.

**Table 4.** Partial Correlations Controlling for Amplitude Sensitivity (*N* = 64)

	2	3	4	5	6	7	9
1. Reading speed (wpm)	.309*	.061	.205	.294*	-.101	.213	.021
2. Reading accuracy (raw)		-.538**	.144	.167	.097	.196	-.048
3. Reading comprehension (raw)			-.276*	.042	-.270*	.369**	-.025
4. Phoneme elision (raw)				.022	-.117	-.289*	.143
5. Nonword repetition (raw)					-.114	.447**	-.029
6. RAN seconds						.075	.004
7. Vocabulary (raw)							.070
8. Amplitude *d*’							

*Note*. wpm = words per minute. *p < .05. **p < .01.

**Table 5.** Partial Correlations Controlling for Tone Frequency Sensitivity (*N* = 64)

	2	3	4	5	6	7	8
1. Reading speed (wpm)	.315*	.059	.155	.302*	-.101	.260*	.189
2. Reading accuracy (raw)		-.539**	.136	.169	.096	.208	.058
3. Reading comprehension (raw)			-.268*	.041	-.270*	.348**	-.005
4. Phoneme elision (raw)				.011	-.114	-.352**	-.219
5. Nonword repetition (raw)					-.114	.445**	.066
6. RAN seconds						.067	-.009
7. Vocabulary (raw)							0.340**
8. Vocabulary (raw)							

*Note*. wpm = words per minute. *p < .05. **p < .01.

To explore the relationship between the non-speech sensitivity measures and the three outcome measures of reading (reading speed, reading accuracy, and comprehension), three multiple regression models were produced with vocabulary as a control in Step 1 and both non-speech sensitivity measures in Step 2. Vocabulary was used in Step 1 because previous studies (e.g., Muter, Hulme, Snowling, & Stevenson, 2004; Webb, 2005) have shown the measure to be related to reading skills. When reading speed was the criterion variable, the non-speech measures did not contribute significant variance above vocabulary. Although a model including only vocabulary had an *adjusted* *R*^2^ = 5.3% and resulted in a significant model, *F*(1, 62) = 4.51, *p* = .04, amplitude and tone frequency at Step 2 accounted for no additional significant variance producing a model that had an *adjusted* *R*^2^ = 3.3%, *F*(3, 60) = 1.72, *p* = .17. Table 6 summarises the contributions of each measure.

**Table 6.** Multiple Regression Model With Reading Speed as an Outcome Variable, Vocabulary in Step 1 and Amplitude and Tone Frequency Sensitivity in Step 2

	*B*	*SE B*	β
Step 1			
Vocabulary (raw)	0.570	0.268	.260
Step 2			
Vocabulary (raw)	0.484	0.289	.221
Amplitude *d*’	6.722	7.782	.114
Tone frequency *d*’	0.383	8.174	.006

Note. *R*^2^ = .07 for Step 1, Δ*R*^2^ = .01 for Step 2 (*p*s = .69 ). *p < .05. **p < .01.

When reading accuracy was used as the criterion variable, neither vocabulary nor the non-speech sensitivity measures produced a significant model. As summarised in Table 7, vocabulary in Step 1accounted for 2.6% of the variance (*adjusted* *R*^2^), *F*(1, 62) = 2.70, *p* = .11, and amplitude and tone frequency in Step 2 accounted for no additional significant variance resulting in an overall model that was non-significant, *F*(3, 60) = 0.96, *p* = .42.

**Table 7.** Multiple Regression Model With Reading Accuracy as an Outcome Variable, Vocabulary in Step 1 and Amplitude and Tone Frequency Sensitivity in Step 2

	B	SE B	β
Step 1			
Vocabulary (raw)	0.143	0.087	.204
Step 2			
Vocabulary (raw)	0.149	0.094	.213
Amplitude *d*’	-0.271	2.539	-.014
Tone frequency *d*’	-1.312	2.667	-.062

Note. *R*^2^ = .042 for Step 1, Δ*R*^2^ = -.002 for Step 2 (*p*s = .88 ). *p < .05. **p < .01.

Finally, when comprehension was the criterion variable, vocabulary alone produced a significant model, accounting for 10.5% of the variance (adjusted *R*^2^), *F*(1, 62) = 8.43, *p* < .01. The non-speech measures, as summarised in Table 8, did not contribute significant additional variance above vocabulary, *F*(3, 60) = 3.23, *p* = .03.

**Table 8.** Multiple Regression Model With Comprehension as an Outcome Variable, Vocabulary in Step 1 and Amplitude and Tone Frequency Sensitivity in Step 2

	B	SE B	β
Step 1			
Vocabulary (raw)	0.256	0.088	.346**
Step 2			
Vocabulary (raw)	0.294	0.095	.397**
Amplitude *d*’	-2.797	2.549	-.140
Tone frequency *d*’	-1.141	2.678	.051

Note. R^2^ = .12 for Step 1, ΔR^2^ = .02 for Step 2 (*p*s = .51 ). *p < .05. **p < .01.

## Discussion

The aim of this study was to investigate the role of the two acoustic components of suprasegmental information in four-syllable words using non-speech stimuli. Participants completed a matching task in which the auditory sequence at the beginning of the pair represented either the amplitude or the tone frequency pattern of a word, and the target was a visually presented four-syllable word. This cross-modal task allowed for a detailed study of both the response times to the pairs and also the sensitivity to detecting these two components of suprasegmental information in words. Moreover, measures of participants’ reading skills were also recorded so that the relationship between reading and sensitivity could be investigated.

In using reaction time and detection sensitivity, the study could highlight different aspects of the suprasegmental cues of words in reading. For response times, tone frequency was faster than amplitude and, for lexical stress patterns, items where the primary stress was in the second syllable were faster than those where the pattern had a primary stress in the third syllable although in sensitivity, as measured by *d*’, there was no significant difference between the amplitude and tone frequency pairs. The fact that tone frequency responses were faster would be in line with the speech perception research that indicated the importance of frequency in the perception of stress patterns (Lieberman, 1960; Mcclean & Tiffany, 1973; Morton & Jassem, 1965; Turk & Sawusch, 1996).

The by-items difference in response time by stress position, where there were faster response times for words with a primary stress on the second syllable compared to words with a primary stress on the third syllable, might suggest that as participants read the target words, they initiated lexical lookups in the same way that the participants in the study by Cutler and Norris (1988) are proposed to have done for auditory stimuli. When reading from left to right, participants would encounter primary stress earlier were it on the second syllable rather than the third syllable and so initiate a lookup earlier with the u1uu word category. This would result in a participant arriving at a matching decision earlier for u1uu words compared with 2u1u words.

However the way participants responded to the matching task itself might suggest two other possible interpretations. The first is that all syllables in the target word could have been processed and then a decision was made. However, it is likely that the response times to both categories of word would then be similar because the lexical processing in both cases would have been completed at the end of the word. The second is that, having been cued to judge whether the stress was on the second or third syllable, participants searched until they could accept or reject the target by starting from the left and moving right, engaging in the syllables of the target word as they went. In doing so, they would have sufficient information to make this decision by the second syllable in the u1uu words and by the third syllable in the 2u1u words. This searching strategy would look like the lookup interpretation that is in line with Cutler and Norris (1988) but would not require as much linguistic processing. Further research would be required to disambiguate the two possible interpretations. However, one prediction from the searching strategy interpretation would be that the response time would be quicker than that found in either a typical lexical decision or naming tasks using the target word items. This is because there would only be a partial processing of the target word before a decision is made. However, the behavioral data from the ELP (Balota et al., 2007) runs counter to this prediction. For lexical decision and naming, the mean response times to the u1uu and 2u1u target words were below 800 ms (for u1uu 696.9 ms and 673.7 ms, respectively, and for 2u1u 707.5 ms and 665.5 ms, respectively) and so considerably shorter than the 2,000 ms, and above, response times found for the matching task reported in this study.

In terms of associations with reading skills, tone frequency played little role in the relationships between the two while amplitude sensitivity was related significantly to vocabulary. Furthermore, given the pattern of changes between the zero-order correlations and the partial correlations, it appeared that sensitivity to amplitude may play a role in reading speed and vocabulary. However, the role amplitude sensitivity may play was not independent of vocabulary. It may be that the sensitivity to non-speech amplitude information indexes some retrieval aspect of word knowledge. This finding would be in line with other non-speech lexical studies, notably the amplitude modulation studies (e.g., Goswami et al., 2002). It may further be that early in reading, and for children with reading difficulties, lexical stress information plays a more direct role. However in relation to skilled adult readers and lexical stress sensitivity, the impact is mediated by other processes that make up fast and accurate skilled reading.

Regarding models of reading (e.g., Coltheart et al., 2001; Perry et al., 2010; Rastle & Coltheart, 2000), the findings lend support to the idea that for known words, there is a reliance on a lexical store for suprasegmental information and that a sensitivity to this information is not directly related to reading but indirectly through other processes. The use of text, rather than pictures (e.g., Schiller et al., 2004, 2006) or another form of target, such as a word fragment (Cooper, Cutler, & Wales, 2002), was so that a reading process would be elicited rather than a word production process. Moreover, participants were not required to name aloud the target words in the matching task. However, there is some commonality between spoken word production and reading (e.g., Indefrey & Levelt, 2004). Presumably once a word is identified then the processes to naming a word are similar whether the word is from text or from another source, including a participant’s own internally generated thoughts. The correlational analyses in this study suggested that, at least for amplitude sensitivity, suprasegmental information had an indirect relationship to reading through vocabulary. However, vocabulary knowledge is involved in speech production (Dell, 1990; Jescheniak et al., 2009) and so an alternative interpretation might be that the association between this measure and amplitude sensitivity may be due to word production processes rather than reading.

There are some key differences in the non-speech auditory measures used here and those used in amplitude modulation or rise time studies (e.g., Hämäläinen, Leppänen, Torppa, Müller, & Lyytinen, 2005). First, in terms of the stimuli, in this study the non-speech stimuli were sounds of a particular consistent duration and amplitude whilst in rise time studies, sounds may change their amplitude either sharply or softly and a certain number of times in a stimulus, as an analogue to changes in vowel stress (Goswami et al., 2002). Second, the measure used in studies such as Hämäläinen et al. (2005) was a measure of acoustic discrimination where two types of sound may be presented and the participant was required to discriminate between them. It might be that sensitivity to discrimination of acoustic information is associated with reading but sensitivity to matches in acoustic information and words is not as strongly associated.

Although the non-speech cues were based around the way suprasegmental information may be conveyed in the acoustic signal and the choice of amplitude and tone frequency stimuli were in line with what may be expected of speech signals, as studies mentioned in the Introduction section used similar ranges, it may be that the non-speech stimuli were too far removed from speech. For example in speech, although /*ab*/ and /*ba*/ are segmentally mirrored, they do not have mirrored frequency patterns (Shattuck & Klatt, 1976) whereas, when represented as amplitude or frequency non-speech tones, high-low or low-high are mirrors of each other. The abstract nature of the cues may have resulted in somewhat muted findings in detection sensitivity. Studies, such as by Rosen and Eva (2001), have shown that speech has particular properties that are not always able to be reflected in non-speech stimuli. Using stimuli more closely based on speech or derived from speech low pass filtering (e.g., Wood & Terrell, 1998) may allow further investigation of aspects relating to accuracy of speech and non-speech stimuli and text targets in future research of this nature.

The study here did not feature duration as a manipulation, although syllables vary in their duration in spoken English, and as the role of syllable duration information in speech perception is still unclear (Plag et al., 2011), further research may help explore the role sensitivity to duration of syllables may have in facilitating reading speed, accuracy, and comprehension. It may be that sensitivity to syllable duration in language, as part of the signals in lexical stress, helps proficiency in these two aspects of reading.

There are some limitations in the generalizability of the findings. First, the study only used four-syllable words and this was to provide enough information for a matching judgment and also to address any potential default effects. As noted in the Introduction section, priming studies of two-syllable words suggest cognition may treat words of other sizes differently (Schiller et al., 2004). However Jared and Seidenberg (1990), using a naming task, would suggest that there may be commonalities in how monosyllabic words and multisyllabic words are processed by systems involved in reading. Given the findings in this study, an exploration of other word stimuli with different lengths may help develop this area of research.

The use of non-speech stimuli raises this issue of how comparable the stimuli were across the two conditions we used to represent components of amplitude and tone frequency (e.g., whether the differences in amplitude provide the same level of sensitivity to target words as the differences in tone frequency). This may give rise to the patterns of sensitivity seen in the Results section. Further studies varying the levels of amplitude and frequency in the stimuli may help explore whether differences in stimuli affect the sensitivity to the prosodic contour.

In terms of the word lists, it was not possible to control for the orthographic correlates of the suprasegmental stress patterns: For example, endings of words may have offered cues that would not have required a lexical lookup. Based on data from the ELP (Balota et al., 2007), irrespective of lexical stress placement, there are 838 possible word endings for four-syllable words (i.e., the last syllable). Of these, 828 word endings occur in less than 2% of English words (together account for around 50.52% of all four-syllable word endings). The remaining 10 word endings together account for around 49.48% of the word endings. In descending order, with approximate proportions in brackets, these are -*ing* (e.g., *classifying* [11.12%]), -*ly* (e.g., *attentively* [8.86%]), -*tion* (e.g., *population* [5.57%]), -*ses* (e.g., *paradises* [4.67%]), -*ed* (e.g., *punctuated* [4.6%]), -*tions* (e.g., *reputations* [3.84%]), -*ble* (e.g., *questionable* [3.15%]), -*ness* (e.g., *impoliteness* [3.06%]), -*ty* (e.g., *activity* [2.59%]), and -*y* (e.g., *ascendancy* [2%]). However, there is little crossover in the word endings of the two groups of words in this study. In the 2u1u word pattern list, the ending -tion predominated (in eight of the 12 words). This is also seen in 2u1u words in general where approximately 19.6% of words with this stress pattern end in -tion and this ending is the most frequent. This is in comparison to the u1uu word list where -ity was the most often used in the word list. This ending accounts for approximately 5.5% of four-syllable u1uu words and is the third most frequent. Since the orthography and the stress pattern of a word is likely to be very closely linked (Kelly, 2004), it would not have been possible to match a u1uu and an 2u1u list on word endings. Participants could have used these text cues to derive the suprasegmental stress pattern in the task without the use of the initial cue. However, it might be possible to control for orthographic cues using nonwords and this would be a possible basis for future investigation.

Moreover, there is an underlying assumption that the participants read the words that were presented targets. Yet, whilst there is no explicit evidence that participants did not do so, there was no additional control to establish this. However, we are relatively confident that reading did occur as reading of lexical stimuli is virtually impossible to suppress in skilled-adult samples as tasks like the Stroop (1935) procedure have demonstrated. Moreover, eye tracking studies such as Ashby and Clifton (2005) suggest that in adult readers stress-based information is processed during silent reading. Another potential participant limitation was the assumption that the participants could read all of the words presented. Results from the reading task, although an indirect measure, suggest that these readers were proficient to a high level of ability, on average the standardized reading scores were above the 60th percentile. However, future studies may benefit from a control task involving verifying the participant’s ability to read the target words.

In conclusion, the study aimed to investigate sensitivity to amplitude and tone information. In doing so, it sought to contribute to an understanding of the use of suprasegmental information in cognition when reading. The findings provide support for the assertion that suprasegmental information is involved in reading known words but that in adult readers some of this information, amplitude information, is indirectly involved in reading through vocabulary access.

## Appendix A

**Table A1.** Words Used in the Suprasegmental Matching Task

u1uu	2u1u
activity	corporation
analysis	information
capacity	democratic
community	population
impossible	situation
particular	generation
philosophy	operation
political	scientific
professional	education
relationship	responsible
security	economic
traditional	application

*Note*. u1uu =words where the primary stress was on the second syllable (unstressed-primary-unstressed-unstressed). 2u1u = words with a primary stress on the third syllable (secondary-unstressed-primary-unstressed).
